# Optimum Reaction Conditions for the Synthesis of Selenized *Ornithogalum caudatum* Ait. (Liliaceae) Polysaccharides and Measurement of Their Antioxidant Activity In Vivo

**DOI:** 10.3390/molecules28155929

**Published:** 2023-08-07

**Authors:** Renshuang Sun, Zhongyuan Qu, Chenfeng Ji, Xiaolong Yang, Yiqiao Zhang, Xiang Zou

**Affiliations:** 1 Medical College, Tonghua Normal University, Tonghua 134001, China; 13624355398@163.com; 2 School of Pharmacy, Harbin University of Commerce, Harbin 150076, China; smilejcf001@sina.com (C.J.); yangxiaolong3399@163.com (X.Y.); zyq7729@163.com (Y.Z.)

**Keywords:** *Ornithogalum caudatum* Ait, selenium polysaccharides, response surface methodology, antioxidant activity, D-galactose-induced aging mouse model

## Abstract

This study determined the optimum reaction conditions for synthesizing selenium-containing polysaccharides. Polysaccharide IIA (with the highest yield) from *Ornithogalum caudatum* Ait. (Liliaceae) (OCAPIIA) was extracted and purified. Then, three parameters were selected to optimize the synthesis of selenized OCAPIIA (Se-OCAPIIA) using the Box–Behnken design (BBD) and response surface methodology (RSM). The morphology of Se-OCAPIIA was analyzed by scanning electron microscopy (SEM). The characteristic peaks and the monosaccharide composition of Se-OCAPIIA were evaluated by Fourier-transform infrared spectroscopy and gas chromatography. A D-galactose-induced aging mouse model was established, and the in vivo antioxidant activity of Se-OCAPIIA was measured. The optimal conditions for the synthesis of Se-OCAPIIA were as follows: reaction temperature, 72.38 °C; Na_2_SeO_3_ to OCAPIIA mass ratio, 0.93 g/g; and reaction time, 8.05 h. The selenium content of Se-OCAPIIA obtained using the optimized process was 3.131 ± 0.090 mg/g, close to the maximum predicted value (3.152 mg/g). Se-OCAPIIA contained D-mannose, D-glucose, and D-galactose at a molar ratio of 1.00:0.34:0.88. SEM showed that Se-OCAPIIA was spherical and flocculated. Compared with OCAPIIA, Se-OCAPIIA exhibited two characteristic peaks at 833 and 610 cm^−1^ in the infrared spectrum. Se-OCAPIIA increased catalase, glutathione peroxidase, and superoxide dismutase activities and decreased MDA concentrations in the mouse liver. Moreover, Se-OCAPIIA treatment improved liver morphology, decreased the levels of IL-1β and IL-6, and increased IL-10 concentration. In conclusion, the synthesis of Se-OCAPIIA is effective, simple, and feasible. Se-OCAPIIA demonstrated high antioxidant activity in vivo and is a promising antioxidant and therapeutic agent.

## 1. Introduction

*Ornithogalum caudatum* Ait (OCA) (Liliaceae), a bulbous plant also known as pearl grass, Niaoru flower, Calabash bar, and squill, is native to Southern Africa and was introduced from North Korea to Changbai County, Jilin Province, China in the nineteenth century [1]. It has been cultivated and used medicinally for centuries. In 2000, Dieffenbachia was approved by the Jilin Provincial Drug Administration as traditional Chinese medicine. OCA has ornamental value and antibacterial, anti-inflammatory, antitumor, and other biological activities. This plant is used extensively in China to treat wounds, liver cirrhosis, hepatitis, lung cancer, colon cancer, and other conditions [2,3,4,5]. The main active ingredients of OCA are saponins, flavonoids, polysaccharides, terpenes, alkaloids, and trace elements [6]. OCA effectively treats liver fibrosis and liver cancer by inducing apoptosis, reducing mitochondrial membrane potential and increasing cytochrome C protein expression and caspase-3 activity [7,8]. OCA exhibits antioxidant activity by scavenging superoxide anions, DPPH radicals, and hydroxyl radicals and increasing the activity of antioxidant enzymes [1,9]. In addition, OCA stimulates cellular and humoral immunity [10]. Most of these effects are attributed to polysaccharides in OCA [11].

Selenium is a trace element necessary for several physiological activities and improves immune response and antioxidant and antitumor activities [12,13]. Selenium deficiency predisposes to the development of many diseases. The organic form of selenium has higher bioavailability than the inorganic form because the former is involved in the formation of several proteins [14]. Selenium polysaccharides (SEPs) are organic selenium compounds whose biological activity is stronger than that of either selenium or polysaccharides. Moreover, SEPs are easily absorbed and have little toxicity [15]. Because of these characteristics, SEPs are widely used as antioxidants, immune modulators, and anti-aging and antitumor agents [16].

SEPs have been found in plants and microorganisms, although molar concentrations in these sources are low. In addition, the production and applications of SEPs are limited because the extraction method is not well-established, and the cost of production is high [17,18]. Notwithstanding, the production of SEPs with high biological activity can be achieved by selenization and chemical synthesis [19]. Qin et al. [20] optimized the selenization of *Angelica* polysaccharides. Selenization increased lymphocyte proliferation and the immune-enhancing activity of polysaccharides. Shang et al. [21] discovered that the inhibitory effect of pumpkin SEPs on human breast cancer MDA-MB-231 cells was significantly stronger than that of unselenized polysaccharides. Moreover, SEPs from *Potentilla anserina* L. effectively scavenged O_2_·,·OH, and DPPH·, and the scavenging activity was positively correlated with the degree of selenium substitution [22]. Synthesized SEPs containing selenium and natural polysaccharides can serve as therapeutic agents and functional additives.

This study, for the first time, established the procedure of hot water (90 °C) extraction of OCA polysaccharides and confirmed that the 40% alcohol precipitation phase had the maximum polysaccharide yield. Unlike prior studies, this technique is better suitable for large-scale industrial production with a low preparation cost [1]. Additionally, Box–Behnken design (BBD) and response surface methodology (RSM) were used to establish the optimal synthesis process of a new selenium polysaccharide, Se-OCAPIIA, with a refined polysaccharide of OCA. Moreover, an in vivo animal model was developed for the evaluation of the antioxidant activity of Se-OCAPIIA, which was more trustworthy than prior in vitro antioxidant evaluation methods [23]. The results may accelerate the development and application of Se-OCAPIIA as a functional ingredient in the food industry and a therapeutic agent.

## 2. Results and Discussion

### 2.1. Establishment and Analysis of RSM

Seventeen experiments were designed using RSM and BBD to assess the combined effects of reaction time (X_1_), reaction temperature (X_2_), and Na_2_SeO_3_-to-OCAPII mass ratio (X_3_) on the selenium concentrations of Se-OCAPIIA (Y). Selenium concentrations varied between 2.006 and 3.125 mg/g (Table 1). The optimum extraction conditions and the response of the combined factors were determined using the following quadratic polynomial regression equation: Y = 3.09 + 0.11X_1_ + 0.22X_2_ + 0.11X_3_ − 0.13X_1_X_2_ − 0.075X_1_X_3_ − 0.084X_2_X_3_ − 0.28X_1_^2^ − 0.34X_2_^2^ − 0.067X_3_^2^. Regression and variance analyses were performed to fit the model (Table 2). The significance of the corresponding factors and the interaction between the combined variables were evaluated by the F-value (positively correlated with the effect of the factors) and *p*-value (negatively correlated with the influence of variables or their interactions) [24]. The coefficient of determination (R^2^), adjusted R^2^ (R^2^_adj_), and coefficient of variation (CV%) were calculated to assess model adequacy.

The results showed that the independent variables (X_1_, X_2_, and X_3_), the respective quadratic terms (X_1_^2^ and X_2_^2^), and the interaction X_1_X_2_ had significant effects on the selenium content of Se-OCAPIIA (*p* < 0.001). Similarly, the interaction terms X_1_X_3_ and X_2_X_3_ and the quadratic term X_3_^2^ significantly affected the selenium concentration of Se-OCAPIIA (*p* < 0.05). According to the F-value, the order of influence of factors on the selenium content was X_2_ > X_3_ > X_1_.

The F-value of the model was 83.63, and the *p*-value was <0.0001, suggesting that the predicted quadratic response surface model was statistically significant. The F-value for the “lack of fit” was 5.98, and the *p*-value was not statistically significant (0.0584), implying that the model equation predicted the selenium content in the range of experimental variables. The R^2^ of the RSM model was 0.9908, suggesting a significant relationship between the influencing factors of known variables and the influencing factors of all variables. Furthermore, 99.08% of the variation in selenium levels came from the selected independent variables, demonstrating that the model was highly accurate. R^2^_adj_ was 0.9789, indicating that 97.89% of the variation was distributed among the three factors in the Se-OCAPIIA binding reaction, and less than 2.5% of the variation was not explained by this model. The CV (%) was low (1.68%), demonstrating the high accuracy and good reliability of the experimental values. These results suggested that the model accurately predicted Se-OCAPIIA preparation conditions.

### 2.2. Optimization and Analysis of the Synthesis of Se-OCAPIIA

**Figure 1:**
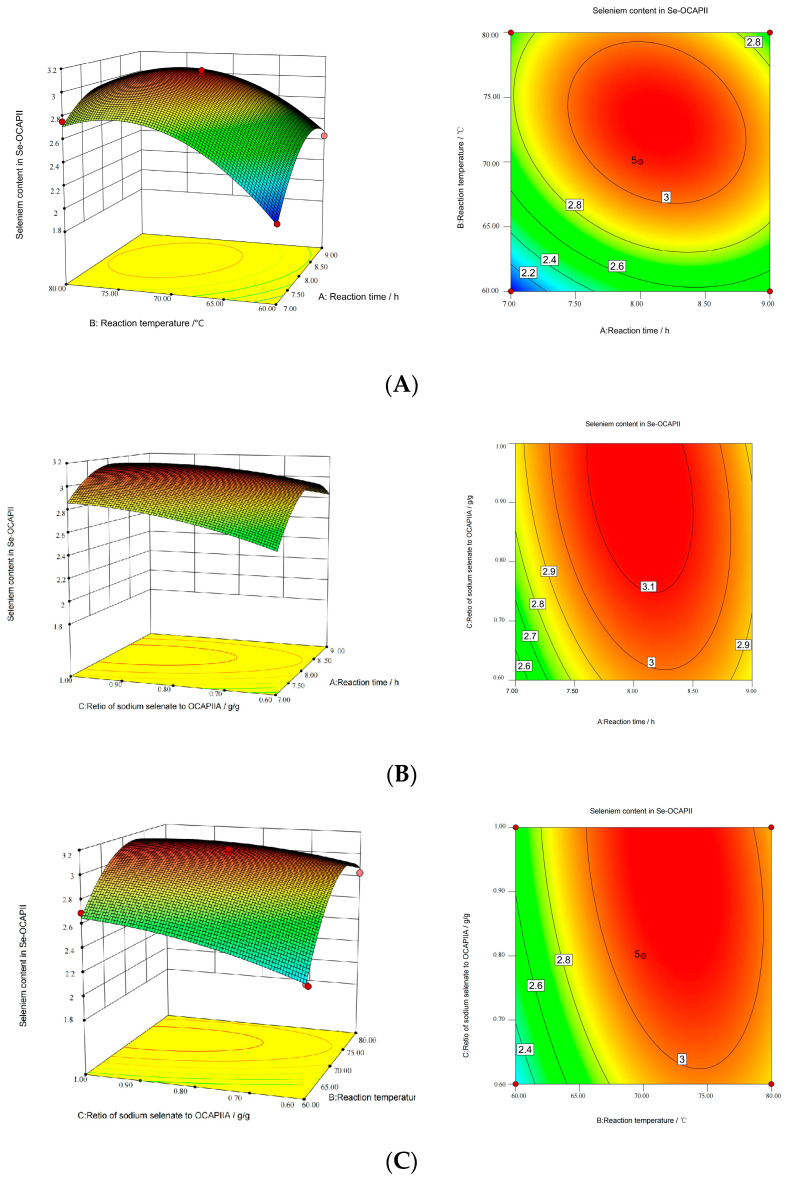
Response surface plots of the influence of various factors on the selenium concentration of Se-OCAPIIA. (**A**) Influence of reaction time and reaction temperature. (**B**) Influence of reaction time and Na_2_SeO_3_-to-OCAPIIA mass ratio. (**C**) Influence of reaction temperature and Na_2_SeO_3_-to-OCAPIIA mass ratio. In response surface plots and diagnostic plots, the change in color from blue to green, to yellow, to red indicates the variation in the selenium content of Se-OCAPIIA from less to more. Contour lines are also colored blue, green, yellow, or red, representing the variation of the selenium content in Se-OCAPIIA from lower to higher under different experiment conditions, in sequence. Additionally, the points on the same line represent the same selenium content of Se-OCAPIIA.

Response surface plots and diagnostic plots were obtained using Design Expert version 7.0. The effects of X_1_, X_2_, and X_3_ on the selenium content of Se-OCAPIIA are shown in Figure 1A–C. In the response surface plots, the selenium content was determined using two continuous variables, whereas the third variable was given a value of 0. There was a conspicuous peak in each plot, indicating that the optimal value of the influencing factors was within the range of experimental values. Selenium concentration initially increased with the increase in reaction temperature and reaction time and then decreased. When reaction time or reaction temperature was fixed, selenium content increased with the Na_2_SeO_3_-to-OCAPIIA mass ratio. The factors with the strongest influence were reaction temperature, Na_2_SeO_3_-to-OCAPIIA mass ratio, and reaction time.

According to the regression model and model fitting, the optimum reaction conditions for the synthesis of Se-OCAPIIA were as follows: reaction temperature, 72.38 °C; Na_2_SeO_3_-to-OCAPIIA mass ratio, 0.93 g/g; and reaction time, 8.05 h. The actual reaction temperature was 72 °C, while the two other experimental conditions were the same as predicted by the model. The experiments were performed using three independent samples (10 mg) under the above conditions. The selenium content of Se-OCAPIIA was 3.131 ± 0.090 mg/g. The coincidence rate was 99.33%, indicating that the optimum reaction conditions of the model for the synthesis of Se-OCAPIIA were accurate and reliable.

### 2.3. Physicochemical Properties of Se-OCAPIIA

The obtained Se-OCAPIIA powder was white, solid, flocculent, tasteless, and soluble in warm and hot water, salt water, diluted acid, and alkali solutions. The powder was insoluble in methanol, ethanol, n-butanol, acetone, petroleum ether, and toluene. The brownish-red color in the phenol-sulfuric acid reaction indicated the presence of polysaccharides in the sample.

### 2.4. Monosaccharide Composition of Se-OCAPIIA

**Figure 2:**
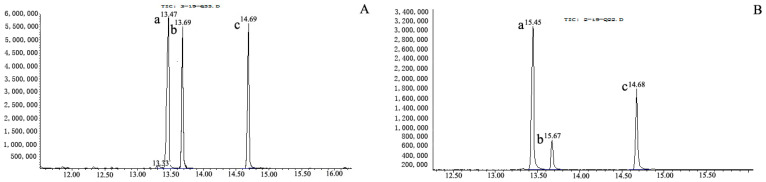
Gas chromatogram of the monosaccharide composition of Se-OCAPIIA. (**A**) Standard monosaccharides—D-mannose (a), D-glucose (b), and D-galactose (c)—were mixed, hydrolyzed, and acetylated to obtain monosaccharide derivatives for gas chromatography analysis. (**B**) The molar mass of each monosaccharide in Se-OCAPIIA was calculated based on the peak areas of a standard monosaccharide mixture.

The monosaccharide composition of Se-OCAPIIA and molar ratios were determined by GC (Figure 2). The monosaccharides were well-separated (Figure 2A). Se-OCAPIIA was composed of D-mannose, D-glucose, and D-galactose (Figure 2B). Based on peak areas, concentrations of the reference and samples, and molar masses, the molar ratio of these monosaccharides was 1.00:0.34:0.88.

### 2.5. Morphologies of OCAPIIA and Se-OCAPIIA

**Figure 3:**
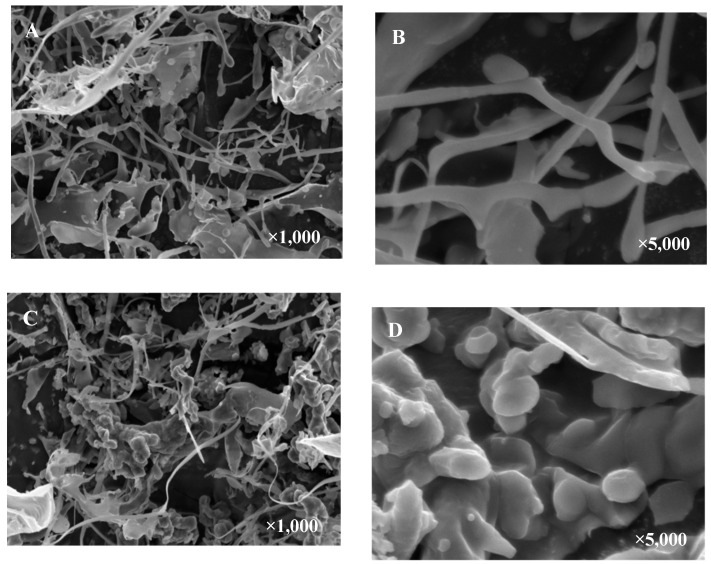
Scanning electron microscopy (SEM) images of OCAPIIA and Se-OCAPIIA. SEM analysis of the morphologies of OCAPIIA (**A**,**B**) and Se-OCAPIIA (**C**,**D**). OCAPIIA appeared as threads and sheets, while Se-OCAPIIA was spherical and flocculated, probably because of the shortening of the polysaccharide chain and partial crystallization caused by selenium.

The morphologies of OCAPIIA and Se-OCAPIIA were analyzed by SEM (Figure 3). OCAPIIA appeared as threads and sheets. After selenization, Se-OCAPIIA became spherical and flocculated, probably because of the shortening of the polysaccharide chain and partial crystallization caused by selenium.

### 2.6. FT-IR Analysis of OCAPIIA before and after Selenization

**Figure 4:**
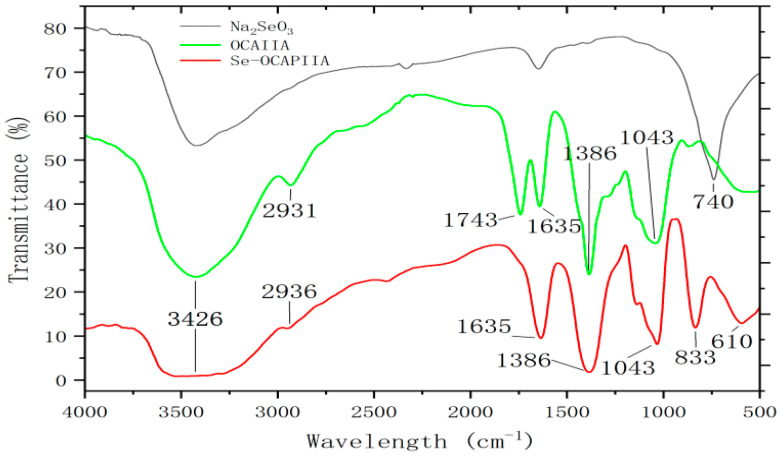
Fourier-transform infrared spectra of Se-OCAPIIA and OCAPIIA. The red, green, and black lines represent Se-OCAPIIA, OCAPIIA, and Na_2_SeO_3_. The absorption peaks of -OH and -CH_2_ are located at 3426 and 2931–2936 cm^−1^, respectively. The absorption peaks of C-O in the sugar ring are located at 1043 and 1635 cm^−1^. The absorption peak of -CH_2_ is located at 1386 cm^−1^. The spectra at 833 and 610 cm^−1^ may correspond to Se-O and Se-OH.

The infrared spectra of OCAPIIA before and after selenization are shown in Figure 4. Selenization did not change the chemical structure of OCAPIIA. In addition, a strong stretching vibration of -OH with a broad absorption peak was located at approximately 3426 cm^−1^. The presence of an intramolecular hydrogen bond increased the peak intensity and width of the stretching vibration. The small peak at 2931–2936 cm^−1^ corresponded to the C-H stretching vibration of -CH_2_. The peak at 1635 cm^−1^ corresponded to the asymmetric stretching of C-O in the sugar ring. The stretching vibration of -CH_2_ and Se-O-H was located at 1386 cm^−1^. The stretching vibration of C-O in the sugar ring was located at 1043 cm^−1^. Se-OCAPIIA had two characteristic peaks at 833 cm^−1^ and 610 cm^−1^. The stretching vibration of -Se-O and the out-of-plane bending vibration of Se-O-H were located at 833 and 610 cm^−1^, respectively [25,26,27,28].

### 2.7. Antioxidant Effect of Se-OCAPIIA In Vivo

**Figure 5:**
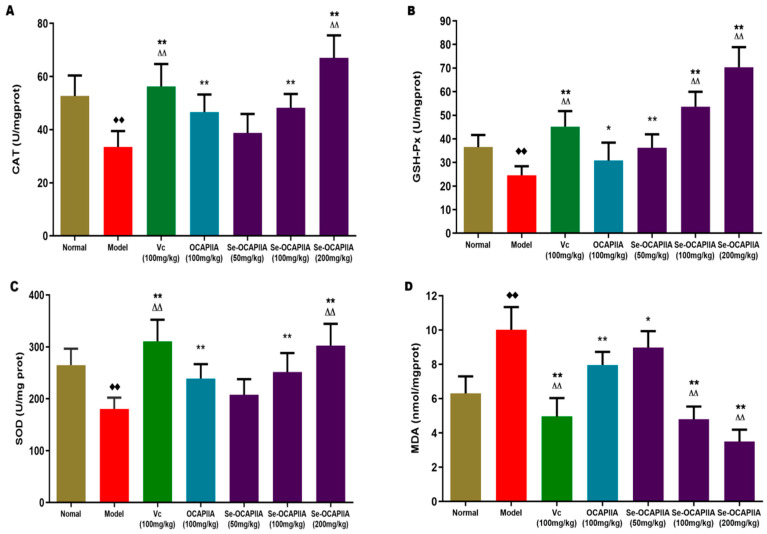
Antioxidant activity of Se-OCAPIIA in a mouse model of aging induced by D-galactose. CAT: catalase; GSH-Px: glutathione peroxidase; SOD: superoxide dismutase; MDA: malondialdehyde; Vc: vitamin C. Compared with normal mice, activities of CAT, GSH-Px, and SOD in the liver of D-gal-treated mice decreased, while MDA level increased dramatically (*p* < 0.01). In turn, Se-OCAPIIA (100 and 200 mg/kg), OCAPIIA (100 mg/kg), and Vc (100 mg/kg, positive control) markedly increased the activities of CAT and SOD (*p* < 0.01). Activities of CAT and SOD in the Se-OCAPIIA (200 mg/kg) and Vc (100 mg/kg) groups were higher than that in the OCAPIIA (100 mg/kg) group (*p* < 0.01) (**A**,**C**). Additionally, Se-OCAPIIA (50-200 mg/kg), OCAPIIA (100 mg/kg), and Vc (100 mg/kg) markedly increased GSH-Px activity and decrease MDA level (*p* < 0.05 or *p* < 0.01). GSH-Px activity in the Se-OCAPIIA (100-200 mg/kg) and Vc (100 mg/kg) groups were higher than that in the OCAPIIA (100 mg/kg) group (*p* < 0.01). On the contrary, MDA in the Se-OCAPIIA (100-200 mg/kg) and Vc (100 mg/kg) groups were lower than that in the OCAPIIA (100 mg/kg) group (*p* < 0.01) (**B**,**D**). These results indicated that Se-OCAPIIA (100–200 mg/kg) and its lead compound, OCAPIIA (100 mg/kg) revealed excellent antioxidant effects in D-gal model mice (*p* > 0.05). * *p* < 0.05, ** *p* < 0.01 vs. the D-galactose group; ^∆∆^ *p* < 0.01 vs. the OCAPIIA group; ^◆◆^ *p* < 0.01 vs. the control group.

Oxidative stress contributes to cardiovascular and neurodegenerative diseases, cancer, and chronic kidney disease [29]. Reactive oxygen species (ROS) produce lipid peroxidation products, including cytotoxic MDA. MDA levels reflect the degree of oxidative damage in tissues. The levels of antioxidant enzymes CAT, GSH-Px, and SOD determine the body’s ability to remove ROS [30,31]. Natural polysaccharides and their derivatives have high antioxidant activity in vivo and in vitro [32]. Moreover, the long-term administration of D-galactose increases ROS production, decreases the activity of antioxidant enzymes in various organs, and generates superoxide anions and other oxidation products, resulting in cellular damage and multiorgan dysfunction. Thus, D-galactose is used to assess the anti-aging and antioxidant effects of natural compounds in animal models [33]. This study determined the antioxidant effect of Se-OCAPIIA and its lead compound, OCAPIIA, in vivo in a mouse model of aging induced by D-galactose and measured MDA levels and the activities of CAT, GSH-Px, and SOD in the mouse liver (Figure 5).

As an enzyme scavenger, CAT converts cytotoxic H_2_O_2_ into molecular oxygen and water, protecting cells from ROS-induced oxidative damage [34]. D-galactose decreased CAT activity, while Se-OCAPIIA (100 and 200 mg/kg), OCAPIIA (100 mg/kg), and vitamin C (100 mg/kg) reversed this effect (Figure 5A). In addition, the ability of 200 mg/kg Se-OCAPIIA and 100 mg/kg vitamin C to restore CAT activity was higher than that of 100 mg/kg OCAPIIA.

GSH-Px is an important component of the peroxidase system. Selenium catalyzes the conversion of GSH into GSSG, transforming toxic peroxides into non-toxic hydroxyl compounds, thus protecting cells from oxidative damage [35]. D-galactose decreased GSH-Px activity, and OCAPIIA, Se-OCAPIIA, and vitamin C reversed this effect (Figure 5B). Furthermore, the abilities of Se-OCAPIIA (100 and 200 mg/kg) and vitamin C to reverse the effect of D-galactose were higher than that of OCAPIIA (100 mg/kg). Since selenocysteine was present in the active site of GSH-Px, Se-OCAPIIA treatment increased selenium concentrations in the body, enhancing GSH-Px activity. These results indicated that selenization increased the antioxidant effect of OCAPIIA.

SOD plays a crucial role in redox homeostasis by controlling ROS damage and ROS signaling [36]. D-galactose notably decreased SOD activity in mice (*p* < 0.01) (Figure 5C), and this effect was reversed by Se-OCAPIIA (100 and 200 mg/kg), OCAPIIA (100 mg/kg), and vitamin C (100 mg/kg). In addition, the ability of Se-OCAPIIA (200 mg/kg) to reverse the effect of D-galactose was higher than that of OCAPIIA (100 mg/kg). These findings indicated that Se-OCAPIIA and its lead compound, OCAPIIA, reversed the effect of D-galactose on SOD activity.

MDA is the main byproduct of membrane lipid peroxidation and is thus used as a marker of oxidative damage [37]. D-galactose increased MDA concentrations in the mouse liver (Figure 5D), while all tested polysaccharides and vitamin C reduced this effect (Figure 5D). Moreover, the scavenging effect of Se-OCAPIIA (100 and 200 mg/kg) on MDA was much stronger than that of OCAPIIA. These results proved that Se-OCAPIIA increased antioxidant activity in D-galactose-treated mice, and selenization increased the antioxidant activity of OCAPIIA in vivo.

### 2.8. Effect of Se-OCAPIIA on Serum Cytokine Levels

ROS activates inflammatory factors, including NF-κB, PI3K, and MAPK, and induces the expression of pro-inflammatory cytokines such as TNF-α, IL-1β, and IL-6, leading to chronic inflammation and accelerating aging [38,39,40]. IL-1β, a major pro-inflammatory mediator, is implicated in host response and resistance to pathogens and the development of chronic diseases and acute tissue injury [41]. IL-6 is produced in response to infections and tissue injury and impairs host defenses by stimulating acute phase responses, hematopoiesis, and immune reactions. IL-6 is upregulated during inflammation. Furthermore, the long-term production of IL-6 induces chronic inflammation and autoimmunity. IL-6 activates JAK1 and JAK2. JAK proteins phosphorylate conserved tyrosine residues in the cytoplasmic domains of receptors (gp130, LIFR, and IL-27Rα) [42,43]. IL-10 is an immunomodulatory cytokine that decreases the production of inflammatory cytokines and ROS. Aging is associated with the upregulations of IL-1β and IL-6 and the downregulation of IL-10 [44].

**Figure 6:**
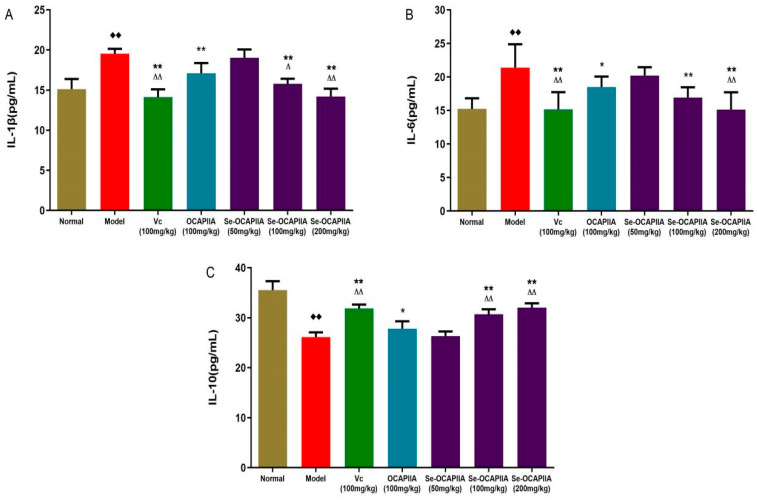
Effect of Se-OCAPIIA on the serum levels of IL-1, IL-6, and IL-10 in aging mice. Vc: vitamin C. The serum levels of pro-inflammatory factors IL-1β and IL-6 were markedly higher in aging mice than in normal mice (*p* < 0.01). However, 100–200 mg/kg Se-OCAPIIA significantly reduced the serum levels of IL-1β and IL-6 in aging mice (*p* < 0.01). At a dose of 100 mg/kg, serum IL-1β level was lower in the Se-OCAPIIA group than in the OCAPIIA group (*p* < 0.05), while serum IL-6 level was slightly lower in the former (*p* > 0.05) (**A**,**B**). The serum level of the anti-inflammatory factor IL-10 was significantly lower in aging mice than in normal mice (*p* < 0.01), while 100–200 mg/kg Se-OCAPIIA noticeably increased the serum level of IL-10 in aging mice (*p* < 0.01). At 100 mg/kg, serum IL-10 was strikingly higher in the Se-OCAPIIA group than in the OCAPIIA group (*p* < 0.05) (**C**). These results suggest that Se-OCAPIIA improves oxidative stress and reduces inflammatory responses in aging mice. * *p* < 0.05, ** *p* < 0.01 vs. the D-galactose-treated group; ^∆^ *p* < 0.05, ^∆∆^ *p* < 0.01 vs. the OCAPIIA group; ^◆◆^ *p* < 0.01 vs. the control group.

Galactose increased the serum levels of IL-1β and IL-6 and decreased the serum level of IL-10, while Se-OCAPIIA (100 and 200 mg/kg), OCAPIIA (100 mg/kg), and vitamin C reversed these effects (Figure 6). These results indicated that Se-OCAPIIA reduced oxidative stress by improving inflammatory responses.

### 2.9. Effect of Se-OCAPIIA on the Morphology of Liver Tissue

**Figure 7:**
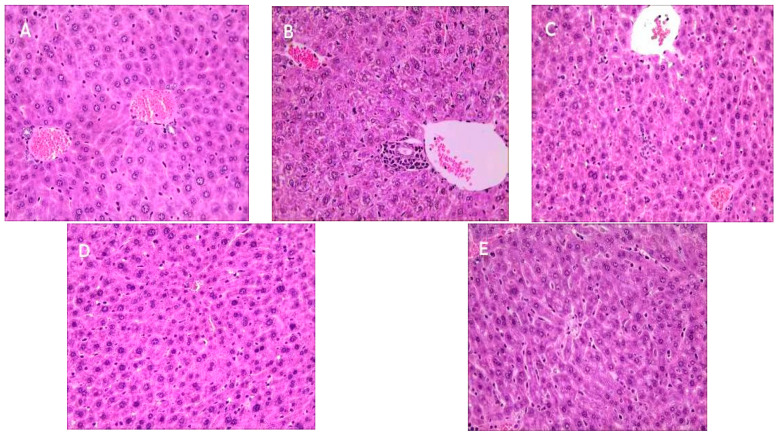
Effects of Se-OCAPIIA on the morphology of liver tissue in aging mice (100×). (**A**) Control. (**B**) D-galactose. (**C**) OCAPIIA (100 mg/kg). (**D**) Se-OCAPIIA (100 mg/kg). (**E**) Vitamin C (100 mg/kg). Hematoxylin–eosin staining showed that OCAPIIA and Se-OCAPIIA improved liver morphology in D-galactose-treated mice: hepatocytes and hepatic cords were well organized, cell infiltration decreased, and the nuclear membrane was well-defined.

To evaluate the protective effects of OCAPIIA and Se-OCAPIIA on the liver of aging mice, histopathological changes in the liver were observed by light microscopy. The morphology of the liver cells of control mice was normal, with uniform size and HE staining. Hepatocytes and hepatic cords were well organized, and cellular margins were well-defined. Furthermore, hepatocytes in control mice had a typical perivascular distribution (Figure 7A). In contrast, the liver tissue of D-galactose-treated mice was characterized by a disordered arrangement of hepatocyte cords, as well as liver cells with uneven distribution, irregular shape, ill-defined margins, and swelling. Vacuoles and inflammatory cell infiltration were observed around the vessels (Figure 7B). OCAPIIA and Se-OCAPIIA reversed these changes (Figure 7C–E).

## 3. Materials and Methods

### 3.1. Samples

*Ornithogalum Caudatum* Ait. was provided by Jilin Huyanmainianqing Biotechnology Co., LTD (Changchun, China) and was identified by Professor Qu Zhongyuan from the Harbin University of Commerce.

### 3.2. Reagents

The reagents included DEAE-52 cellulose (GE Whatman Co., Ltd., Maidstone, UK); papain (Tianjin Guangfu Fine Chemical Research Institute, Tianjin, China); sodium selenite (Tianjin Zhiyuan Chemical Reagent Co., Ltd., Tianjin, China); Viskase dialysis membrane (Viskase Corp., Lombard, IL, USA); D-mannose, D-galactose, and D-glucose (Sinopharm Chemical Reagent Co., Ltd., Shanghai, China); vitamin C (purity: ≥99.0%; Shanghai Aladdin Biochemical Technology Co., Ltd., Shanghai, China); kits for the detection of IL-1β, IL-6, and IL-10 (Jiangsu Kte Biotech Co., Ltd., Yancheng, China); kits for the detection of catalase (CAT), malondialdehyde (MDA), superoxide dismutase (SOD), and glutathione peroxidase (GSH-Px) (Nanjing Jiancheng Bioengineering Institute, Nanjing, China); and ethanol, acetone, petroleum ether, n-butyl alcohol, chloroform, concentrated sulfuric acid, phenol, ammonium hydroxide, and hydrochloric acid (Tianjin Fuyu Fine Chemical Co., Ltd., Tianjin, China). All reagents were of analytical grade.

### 3.3. Instruments

Instruments included the gas chromatograph (6890 N, Agilent Technologies Inc., Palo Alto, CA, USA); atomic fluorescence spectrometer (AFS-9230, Beijing Jitian Instrument Co., Ltd., Beijing, China); oven-controlled oscillator (KDZ, Shanghai Yiheng Technology Co., Ltd., Shanghai, China); rotary evaporator (N-1000, Shanghai Ailang Instrument Co., Ltd., Shanghai, China); freeze-dryer (LGJ-50C-H, Beijing Sihuan Scientific Instrument Factory, Beijing, China); UV/VIS spectrophotometer (T6, Beijing Puxi General Instrument Co., Ltd., Beijing, China); low-speed centrifuge (LD4-2A, Beijing Jingli Centrifuge Co., Ltd., Beijing, China); scanning electron microscope (S-3400N, Hitachi Corporation, Tokyo, Japan); and Fourier-transform infrared (FT-IR) spectrometer (FT-IR 650, Tianjin Gangdong SCI & TECH Development Co., Ltd., Tianjin, China).

### 3.4. Experimental Animals

Male ICR mice (weight: 18–22 g) were purchased from Changchun Yisi Experimental Animal Technology Co., Ltd. (Changchun, China; Quality Certificate No. 202100038308). The animals were housed in solid-bottomed polycarbonate cages under standard conditions, with free access to food (a commercial pelleted diet) and water. The animal studies were approved by the Animal Care and Use Committee of the Engineering Research Center for Natural Antineoplastic Drugs of the Ministry of Education of the Harbin University of Commerce.

### 3.5. Extraction and Purification of OCAPIIA

OCA powder was refluxed with 95% ethanol to remove fat-soluble impurities. Then, the dried OCA residues were extracted in water at 90 °C three times (3 h each time) at a water/sample ratio of 25:1. The solution was filtered and precipitated with 95% ethanol (final density: 40%) for 24 h. The samples were centrifuged at 2500× *g* for 30 min, and precipitated polysaccharides were dissolved in distilled water. Proteins were removed using the Papain-Sevag method, pigments were removed using hydrogen peroxide, and small impurities were removed by dialysis. The solution was dialyzed against running water for 48 h and distilled water for 24 h. Then, OCAPII was obtained and transferred to a DEAE-cellulose column equilibrated with water. Fractions were eluted with distilled water at a flow rate of 1.0 mL/min and collected in test tubes every 5 mL, in a total of 80 tubes (approximately four times the column volume). A standard curve was constructed by plotting absorbance values versus the sample number. The fraction eluted from a single peak was collected and dialyzed against running water for 48 h and distilled water dialysis for 24 h. Then, the purified polysaccharide A (OCAPIIA) was concentrated and lyophilized.

### 3.6. Selenization of OCAPIIA

One gram of OCAPIIA was dissolved in 100 mL of nitric acid solution (0.5–1.0%) to obtain a polysaccharide solution (10 mg/mL). Na_2_SeO_3_ was added to the solution at a ratio of 0.6–1.0 g per g of OCAPIIA, and the reaction was carried out at 60–80 °C. After the reaction, the pH of the solution was adjusted to 5.0–6.0. The solution was centrifuged and dialyzed against running water. Ascorbic acid (40 g/L) was added to the dialysate. Dialysis was interrupted when the red color disappeared. Then, the dialysate was concentrated to 10–20 mL and lyophilized to obtain Se-OCAPIIA.

The extraction conditions were optimized using the BBD. Three factors—reaction time (X_1_), reaction temperature (X_2_), and Na_2_SeO_3_-to-OCAPIIA mass ratio (X_3_)—were selected as independent variables, and the selenium concentration in Se-OCAPIIA was considered a response value (Y) to perform response surface analysis using three factors and three levels and to obtain the optimal parameters for the synthesis of Se-OCAPIIA [45]. The optimal selenium concentration for Se-OCAPIIA (Y) was determined by atomic fluorescence spectrometry (AFS) and RSM. Each independent variable was coded as −1 (low), 0 (intermediate), and 1 (high) (Table 3). The detection parameters of AFS were as follows: photomultiplier tube voltage, 270 V; lamp current, 80 mA; atomizer height, 8 mm; carrier gas (argon) flow rate, 400 mL/min; and shielding gas flow rate, 800 mL/min. The peak areas were measured.

### 3.7. Analysis of the Physical and Chemical Properties of Se-OCAPIIA

The color, physical state, smell, taste, solubility, and other physical properties of Se-OCAPIIA were analyzed. One milligram of Se-OCAPIIA was dissolved in 1 mL of distilled water to obtain a Se-OCAPIIA solution; 1 mL of 6% phenol solution and 5 mL of concentrated sulfuric acid were added to the reaction. The solution was heated to 100 °C for 15 min, and the color change of the solution was observed.

### 3.8. Analysis of the Monosaccharide Composition of Se-OCAPIIA by Gas Chromatography (GC)

A Se-OCAPIIA sample (10 mg) was transferred to an ampoule; 2 mL of trifluoroacetic acid (2 mol/L) was added, and the ampoule was sealed under vacuum. The sample was hydrolyzed at 100 °C for 180 min, dissolved in 1.5 mL of methanol, and dried. This procedure was repeated thrice. One milligram of inositol, 10 mg of hydroxylamine hydrochloride, and 1 mL of anhydrous pyridine were sequentially added to the hydrolyzed sample. The reaction was carried out under vacuum at 90 °C for 30 min. Then, 1 mL of anhydrous acetic acid was added to the sample, and the reaction was carried out for 30 min under the same experimental conditions. The aldononitrile acetate derivatives of Se-OCAPIIA were extracted using chloroform. D-galactose, D-mannose, and D-glucose (10 mg each) were treated the same way as Se-OCAPIIA to obtain their aldononitrile acetate derivatives for GC detection. The following chromatographic parameters were used: HP-5 column, 30 m × 0.32 mm; film thickness, 0.25 μm; detector type, flame ionization; flow rates (in mL/min) (carrier gas [nitrogen], 30; auxiliary gas [air], 450; combustion gas [hydrogen], 40; makeup gas [nitrogen], 0.8; split ratio, 30:1; detector temperature, 250 °C; injection chamber temperature, 280 °C; and injection volume, 1 μL. The temperature gradient was 150 °C for 8 min, 150 °C to 170 °C (5 °C/min) for 5 min, and 170 °C to 200 °C (3 °C/min) for 4 min.

### 3.9. Scanning Electron Microscopy (SEM) Analysis of Se-OCAPIIA

OCAPIIA and Se-OCAPIIA samples were sputter-coated with gold for SEM. The detection conditions were as follows: accelerating voltage, 10 kV; and magnification, 1000–5000×.

### 3.10. FT-IR Analysis

OCAPIIA and Se-OCAPIIA samples (1–2 mg) were ground, mixed with 200 mg of KBr powder, and pressed into pellets. Infrared spectra in the 4000–400 cm^−1^ range were recorded using an infrared spectrometer.

### 3.11. Antioxidant Activity of Se-OCAPIIA In Vivo

Seventy male mice were randomly divided into seven groups (10 mice per group) to receive sterile saline (control and model groups), vitamin C (100 mg/kg/day, positive control), OCAPIIA (100 mg/kg/day, OCAPIIA group), or Se-OCAPIIA (50, 100, or 200 mg/kg/day, Se-OCAPIIA groups). All drugs were diluted with saline solution sterilized by filtration. Mice adapted to the environment for 8 days. After randomization, all mice except those in the normal group were subcutaneously injected with 0.2 mL D-galactose (D-gal) (120 mg/kg/day) in the upper back for 6 continuous weeks. The control group was subcutaneously injected with the same volume of sterile saline [46]. Different dosages of polysaccharides were administered intragastrically for 6 weeks (except in the control group). All mice were weighed 24 h after the last dose. After euthanasia, blood samples were collected from the eyeballs and centrifuged at 2100 *g* for 15 min. The serum concentrations of IL-1β, IL-6, and IL-10 were measured. Liver samples were collected and weighed. MDA concentrations and the enzyme activities of CAT, GSH-Px, and SOD were measured in these samples.

### 3.12. Histopathological Analysis of Liver Tissues

The liver tissues of mice were fixed in a 10% neutral phosphate-buffered formalin solution, dehydrated, embedded in paraffin, sectioned (4-mm thickness), and stained with hematoxylin and eosin (HE). Histopathological changes were observed by light microscopy.

### 3.13. Statistical Analysis

Response surface plots and heat maps were generated and analyzed using Design Experts version 7.0. The experiments were conducted in triplicate, and the results were expressed as means and standard deviations. The significance of differences between means was determined by one-way analysis of variance using SPSS software version 11.5 (SPSS, Inc., Chicago, IL, USA) for Windows.

## 4. Conclusions

The optimal reaction conditions for the synthesis of Se-OCAPIIA based on BBD and RSM were as follows: reaction temperature, 72 °C; Na_2_SeO_3_-to-OCAPIIA mass ratio, 0.93 g/g; and reaction time, 8.05 h. Under these experimental conditions, the selenium concentration in Se-OCAPIIA was 3.131 ± 0.090 mg/g (average of three measurements), close to the maximum predicted value (3.152 mg/g). The coincidence rate was 99.33%. Statistical analysis proved that the RSM model and BBD accurately predicted the optimal conditions for producing Se-OCAPIIA. Se-OCAPIIA was composed of D-mannose, D-glucose, and D-galactose at a molar ratio of 1.00:0.34:0.88. Se-OCAPIIA had higher antioxidant activity than OCAPIIA in vivo. Therefore, Se-OCAPIIA can be used as an antioxidant agent in the food industry and as a therapeutic agent in the clinic. Nonetheless, the industrial production and structural characterization of selenium polysaccharides should be further investigated.

## Figures and Tables

**Table 1:** Box–Behnken design and measurement of the selenium concentration of Se-OCAPIIA.

Run Number	Coded Levels			Selenium Concentration (mg/g)
	X_1_	X_2_	X_3_	
1	8	60	1	2.693
2	8	70	0.8	3.076
3	9	70	1	2.889
4	9	80	0.8	2.663
5	7	70	0.6	2.438
6	8	60	0.6	2.253
7	7	80	0.8	2.754
8	7	70	1	2.766
9	8	70	0.8	3.053
10	9	60	0.8	2.44
11	8	70	0.8	3.081
12	8	70	0.8	3.091
13	8	70	0.8	3.125
14	8	80	0.6	2.833
15	8	80	1	2.936
16	7	60	0.8	2.006
17	9	70	0.6	2.862

**Table 2:** Parameters of the response surface quadratic model.

Source	Sum of Squares	df	Mean Square	F-Value	*p*-Value
Model	1.63	9	0.18	83.63	<0.0001 ^b^
X_1_	0.099	1	0.099	45.85	0.0003 ^b^
X_2_	0.40	1	0.40	186.32	<0.0001 ^b^
X_3_	0.10	1	0.10	46.68	0.0002 ^b^
X_1_X_2_	0.069	1	0.069	31.91	0.0008 ^b^
X_1_X_3_	0.023	1	0.023	10.49	0.0143 ^a^
X_2_X_3_	0.028	1	0.028	13.15	0.0084 ^a^
X_1_^2^	0.33	1	0.33	152.58	<0.0001 ^b^
X_2_^2^	0.49	1	0.49	225.05	<0.0001 ^b^
X_3_^2^	0.019	1	0.019	8.68	0.0215 ^a^
Residual	0.015	7	0.0022		
Lack of fit	0.012	3	0.0041	5.98	0.0584 ^c^
Pure error	0.0028	4	0.0007		
Total correlation	1.64	16			
R^2^	0.9908				
Adjusted R^2^	0.9789				
Adeq Precision (signal-to-noise ratio)	30.478				
Coefficient of variation	1.68				

^a^ Significant at *p* < 0.05; ^b^ significant at *p* < 0.001; ^c^ not significant; df: degrees of freedom.

**Table 3:** Main factors and levels for the extraction of Se-OCAPIIA.

Factor	Level		
	−1	0	1
X_1_: reaction time (h)	7	8	9
X_2_: reaction temperature (°C)	60	70	80
X_3_: Na_2_SeO_3_-to-OCAPIIA mass ratio (g/g)	0.6	0.8	1.0

## Data Availability

No new data were created or analyzed in this study. Data sharing is not applicable to this article.
